# 7-Bromo-1-(4-chloro­phenyl­sulfan­yl)-2-phenyl­naphtho[2,1-*b*]furan

**DOI:** 10.1107/S1600536809052945

**Published:** 2009-12-16

**Authors:** Hong Dae Choi, Pil Ja Seo, Byung Ki Kim, Byeng Wha Son, Uk Lee

**Affiliations:** a Department of Chemistry, Dongeui University, San 24 Kaya-dong Busanjin-gu, Busan 614-714, Republic of Korea; b Department of Molecular Biology, Dongeui University, San 24 Kaya-dong, Busanjin-gu, Busan 614-714, Republic of Korea; c Department of Chemistry, Pukyong National University, 599-1 Daeyeon 3-dong, Nam-gu, Busan 608-737, Republic of Korea

## Abstract

In the title compound, C_24_H_14_BrClOS, the S-bound 4-chloro­phenyl ring is nearly perpendicular to the plane of the naphthofuran fragment [dihedral angle = 83.34 (3)°] and the phenyl ring in the 2-position is rotated out of the naphthofuran plane by a dihedral angle of 15.23 (5)°. The crystal structure is stabilized by aromatic π–π inter­actions between the furan and the central benzene rings of the neighbouring naphthofuran fragments, and between the outer benzene rings of the neighbouring naphthofuran fragments; the centroid–centroid distances within the stack are 3.879 (2) and 3.857 (2) Å. In addition, inter­molecular C—Cl⋯π inter­actions [3.505 (2) Å] between the Cl atom and the 2-phenyl ring are present.

## Related literature

For the crystal structures of similar 7-bromo-2-phenyl­naphtho[2,1-*b*]furan derivatives, see: Choi *et al.* (2009*a* ▶,*b* ▶). For the biological activity of naphthofuran compounds, see: Hagiwara *et al.* (1999 ▶); Hranjec *et al.* (2003 ▶); Mahadevan & Vaidya (2003 ▶).
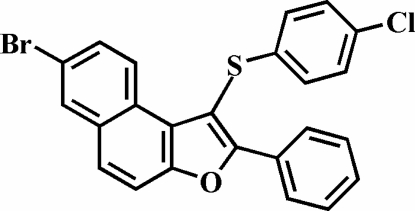


## Experimental

### 

#### Crystal data

C_24_H_14_BrClOS
*M* *_r_* = 465.77
Triclinic, 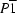

*a* = 8.2479 (3) Å
*b* = 8.3136 (4) Å
*c* = 13.9805 (6) Å
α = 93.530 (2)°
β = 99.317 (2)°
γ = 90.342 (2)°
*V* = 944.07 (7) Å^3^
*Z* = 2
Mo *K*α radiation
μ = 2.44 mm^−1^
*T* = 173 K
0.40 × 0.20 × 0.05 mm

#### Data collection

Bruker SMART APEXII CCD diffractometer
Absorption correction: multi-scan (*SADABS*; Bruker, 2009 ▶) *T*_min_ = 0.560, *T*_max_ = 0.887
16830 measured reflections
4364 independent reflections
3912 reflections with *I* > 2σ(*I*)
*R*_int_ = 0.024

#### Refinement

*R*[*F* ^2^ > 2σ(*F* ^2^)] = 0.026
*wR*(*F* ^2^) = 0.068
*S* = 1.05
4364 reflections
253 parameters
H-atom parameters constrained
Δρ_max_ = 0.37 e Å^−3^
Δρ_min_ = −0.33 e Å^−3^

### 

Data collection: *APEX2* (Bruker, 2009 ▶); cell refinement: *SAINT* (Bruker, 2009 ▶); data reduction: *SAINT*; program(s) used to solve structure: *SHELXS97* (Sheldrick, 2008 ▶); program(s) used to refine structure: *SHELXL97* (Sheldrick, 2008 ▶); molecular graphics: *ORTEP-3* (Farrugia, 1997 ▶) and *DIAMOND* (Brandenburg, 1998 ▶); software used to prepare material for publication: *SHELXL97*.

## Supplementary Material

Crystal structure: contains datablocks global, I. DOI: 10.1107/S1600536809052945/bg2314sup1.cif

Structure factors: contains datablocks I. DOI: 10.1107/S1600536809052945/bg2314Isup2.hkl

Additional supplementary materials:  crystallographic information; 3D view; checkCIF report

## Figures and Tables

**Fig. 1.**
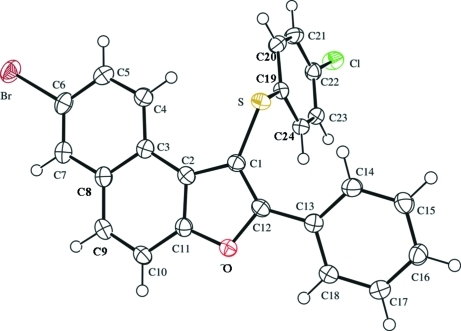
The molecular structure of the title compound with the atom numbering scheme. Displacement ellipsoids are drawn at the 50% probability level.

**Fig. 2.**
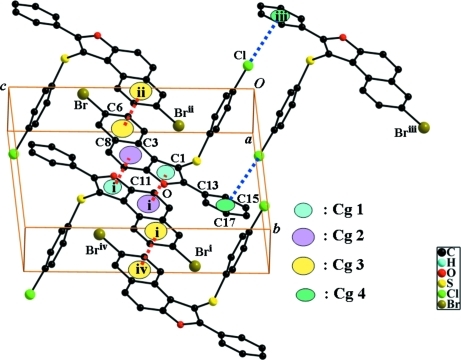
π–π and C—Cl···π interactions (dotted lines) in the crystal structure of the title compound. Cg denotes the ring centroids (see text). [Symmetry codes: (i) - x + 1, - y + 1, - z + 1; (ii) - x + 1, - y, - z + 1; (iii) - x + 1, - y, - z; (iv) x, y + 1, z]

## supplementary crystallographic information

### Comment

Molecules bearing naphthofuran skeleton have attracted considerable interest in view of their biological activity (Hagiwara *et al.*, 1999; Hranjec *et al.*, 2003; Mahadevan & Vaidya, 2003). As a part of our continuing studies of the effect of side chain substituents on the solid state structures of 7-bromo-2-phenylnaphtho[2,1-b]furan analogues (Choi *et al.*, 2009*a, b*), we report the crystal structure of the title compound (Fig. 1).

The naphthofuran unit is essentially planar, with a mean deviation of 0.009 (1) Å from the least-squares plane defined by the thirteen constituent atoms. In the crystal structure, the dihedral angle formed by the plane of the naphthofuran fragment and the 2-phenyl ring (C13–C18) is 15.23 (5)°, and the S-bound 4-chlorophenyl ring makes a diheral angle of 83.34 (3)° with the plane of the naphthofuran fragment. The crystal packing (Fig. 2) is stabilized by two different aromatic π–π interactions within each stack molecules; the first between the furan ring (Cg1) and the central benzene ring (Cg2^i^,  (i):  - *x* + 1,  - *y* + 1,  - *z* + 1) of the neighbouring naphthofuran units [distance; 3.879 (2) Å], and the second between the outer benzene ring (Cg3) and the outer benzene ring (Cg3^ii^,  (ii):  - *x* + 1, - *y*,  - *z* + 1) of the neighbouring naphthofuran units [distance; 3.857 (2)Å] (Cg1, Cg2 and Cg3 are the centroides of the C1/C2/C11/O/C12 furan ring, the C2/C3/C8/C9/C10/C11 benzene ring and the C3–C8 benzene ring, respectively). The molecular packing (Fig. 2) is further stabilized by intermolecular C—Cl···π interactions between the chlorine and the phenyl ring (Cg4), with a C22—Cl···Cg4^iii^ [3.505 (2) Å] (Cg4 is the centroid of the C13–C18 benzene ring,  (iii):  - *x* + 1, - *y*, - *z*).

### Experimental

Zinc chloride (273 mg, 2.0 mmol) was added to a stirred solution of 6-bromonaphthol (446 mg, 2.0 mmol) and 2-chloro-2-(4-chlorophenylsulfanyl)acetophenone (594 mg, 2.0 mmol) in dichloromethane (40 ml) at room temperature, and stirring was continued at the same temperature for 40 min. The reaction was quenched by the addition of water and the organic layer separated, dried over magnesium sulfate, filtered and concentrated in vacuum. The residue was purified by column chromatography (carbon tetrachloride) to afford the title compound as a colorless solid [yield 74%, m.p. 481–482 K;  *R*_f_ = 0.8 (carbon tetrachloride)]. Single crystals suitable for X-ray diffraction were prepared by evaporation of a solution of the title compound in tetrahydrofuran at room temperature.

### Refinement

All H atoms were positioned geometrically and refined using a riding model, with C—H = 0.95 Å for aromatic H atoms and with *U*_iso_(H) = 1.20*U*_eq_(C) for aromatic H atoms.

### Crystal data

**Table:**

C_24_H_14_BrClOS	*Z* = 2
*M**_r_* = 465.77	*F*(000) = 468
Triclinic, *P*1	*D*_x_ = 1.639 Mg m^−^^3^
Hall symbol: -p 1	Mo *K*α radiation, λ = 0.71073 Å
*a* = 8.2479 (3) Å	Cell parameters from 2528 reflections
*b* = 8.3136 (4) Å	θ = 2.3–25.7°
*c* = 13.9805 (6) Å	µ = 2.44 mm^−^^1^
α = 93.530 (2)°	*T* = 173 K
β = 99.317 (2)°	Block, colourless
γ = 90.342 (2)°	0.40 × 0.20 × 0.05 mm
*V* = 944.07 (7) Å^3^	

### Data collection

**Table:**

Bruker SMART APEXII CCD diffractometer	4364 independent reflections
Radiation source: Rotating Anode	3912 reflections with *I* > 2σ(*I*)
HELIOS	*R*_int_ = 0.024
Detector resolution: 10.0 pixels mm^-1^	θ_max_ = 27.6°, θ_min_ = 1.5°
φ and ω scans	*h* = −10→10
Absorption correction: multi-scan (*SADABS*; Bruker, 2009)	*k* = −10→10
*T*_min_ = 0.560, *T*_max_ = 0.887	*l* = −18→18
16830 measured reflections	

### Refinement

**Table:**

Refinement on *F*^2^	Primary atom site location: structure-invariant direct methods
Least-squares matrix: full	Secondary atom site location: difference Fourier map
*R*[*F*^2^ > 2σ(*F*^2^)] = 0.026	Hydrogen site location: inferred from neighbouring sites
*wR*(*F*^2^) = 0.068	H-atom parameters constrained
*S* = 1.05	*w* = 1/[σ^2^(*F*_o_^2^) + (0.0368*P*)^2^ + 0.2823*P*] where *P* = (*F*_o_^2^ + 2*F*_c_^2^)/3
4364 reflections	(Δ/σ)_max_ < 0.001
253 parameters	Δρ_max_ = 0.37 e Å^−^^3^
0 restraints	Δρ_min_ = −0.33 e Å^−^^3^

### Special details

**Table:**

Geometry. All esds (except the esd in the dihedral angle between two l.s. planes) are estimated using the full covariance matrix. The cell esds are taken into account individually in the estimation of esds in distances, angles and torsion angles; correlations between esds in cell parameters are only used when they are defined by crystal symmetry. An approximate (isotropic) treatment of cell esds is used for estimating esds involving l.s. planes.
Refinement. Refinement of F^2^ against ALL reflections. The weighted R-factor wR and goodness of fit S are based on F^2^, conventional R-factors R are based on F, with F set to zero for negative F^2^. The threshold expression of F^2^ > 2sigma(F^2^) is used only for calculating R-factors(gt) etc. and is not relevant to the choice of reflections for refinement. R-factors based on F^2^ are statistically about twice as large as those based on F, and R- factors based on ALL data will be even larger.

### Fractional atomic coordinates and isotropic or equivalent isotropic displacement parameters (Å^2^)

**Table:**

	*x*	*y*	*z*	*U*_iso_*/*U*_eq_	
Br	0.16900 (2)	−0.00651 (2)	0.696104 (13)	0.03934 (7)	
S	0.32781 (5)	0.41585 (5)	0.26003 (3)	0.02372 (9)	
Cl	0.23890 (5)	−0.23943 (5)	0.00792 (3)	0.03551 (11)	
O	0.79044 (13)	0.43650 (13)	0.39137 (8)	0.0236 (2)	
C1	0.52090 (18)	0.40291 (18)	0.33320 (11)	0.0215 (3)	
C2	0.55319 (19)	0.33416 (17)	0.42666 (11)	0.0220 (3)	
C3	0.45931 (19)	0.25242 (17)	0.48592 (11)	0.0226 (3)	
C4	0.2890 (2)	0.21797 (19)	0.46150 (12)	0.0271 (3)	
H4	0.2308	0.2497	0.4015	0.033*	
C6	0.2914 (2)	0.09476 (19)	0.61175 (12)	0.0292 (3)	
C7	0.4563 (2)	0.12282 (19)	0.63870 (12)	0.0286 (3)	
H7	0.5114	0.0886	0.6989	0.034*	
C8	0.5445 (2)	0.20304 (18)	0.57655 (11)	0.0252 (3)	
C9	0.7169 (2)	0.2345 (2)	0.60430 (11)	0.0282 (3)	
H9	0.7712	0.1995	0.6645	0.034*	
C10	0.8055 (2)	0.3132 (2)	0.54683 (11)	0.0267 (3)	
H10	0.9197	0.3355	0.5659	0.032*	
C5	0.2067 (2)	0.1400 (2)	0.52255 (13)	0.0295 (3)	
H5	0.0926	0.1165	0.5047	0.035*	
C11	0.71960 (19)	0.35948 (18)	0.45816 (11)	0.0232 (3)	
C12	0.66752 (19)	0.46116 (18)	0.31452 (11)	0.0225 (3)	
C13	0.72391 (19)	0.52879 (18)	0.23142 (11)	0.0227 (3)	
C14	0.6152 (2)	0.5820 (2)	0.15241 (12)	0.0294 (3)	
H14	0.5004	0.5809	0.1537	0.035*	
C15	0.6734 (2)	0.6363 (2)	0.07257 (12)	0.0304 (4)	
H15	0.5982	0.6708	0.0192	0.037*	
C16	0.8403 (2)	0.6409 (2)	0.06961 (12)	0.0289 (3)	
H16	0.8797	0.6781	0.0146	0.035*	
C17	0.9493 (2)	0.5907 (2)	0.14774 (13)	0.0324 (4)	
H17	1.0641	0.5944	0.1464	0.039*	
C18	0.8923 (2)	0.5350 (2)	0.22773 (12)	0.0281 (3)	
H18	0.9685	0.5007	0.2807	0.034*	
C19	0.30880 (19)	0.22538 (18)	0.19376 (11)	0.0219 (3)	
C20	0.1534 (2)	0.1543 (2)	0.16982 (12)	0.0271 (3)	
H20	0.0622	0.2044	0.1923	0.033*	
C21	0.1313 (2)	0.0107 (2)	0.11338 (12)	0.0295 (3)	
H21	0.0251	−0.0377	0.0965	0.035*	
C22	0.2658 (2)	−0.06179 (19)	0.08176 (11)	0.0257 (3)	
C23	0.4219 (2)	0.0055 (2)	0.10721 (11)	0.0255 (3)	
H23	0.5135	−0.0471	0.0868	0.031*	
C24	0.44322 (19)	0.15046 (19)	0.16274 (11)	0.0241 (3)	
H24	0.5496	0.1986	0.1796	0.029*	

### Atomic displacement parameters (Å^2^)

**Table:**

	*U*^11^	*U*^22^	*U*^33^	*U*^12^	*U*^13^	*U*^23^
Br	0.04043 (12)	0.04369 (12)	0.03854 (11)	−0.00180 (8)	0.01701 (8)	0.01182 (8)
S	0.02033 (18)	0.02376 (19)	0.02618 (19)	0.00233 (14)	0.00094 (14)	0.00188 (14)
Cl	0.0318 (2)	0.0343 (2)	0.0391 (2)	−0.00495 (17)	0.00692 (18)	−0.01079 (17)
O	0.0224 (5)	0.0263 (6)	0.0215 (5)	−0.0027 (4)	0.0017 (4)	0.0017 (4)
C1	0.0211 (7)	0.0209 (7)	0.0220 (7)	0.0008 (6)	0.0022 (6)	0.0002 (5)
C2	0.0248 (8)	0.0188 (7)	0.0218 (7)	0.0015 (6)	0.0027 (6)	−0.0015 (5)
C3	0.0267 (8)	0.0178 (7)	0.0235 (7)	0.0013 (6)	0.0060 (6)	−0.0014 (5)
C4	0.0266 (8)	0.0278 (8)	0.0269 (8)	0.0012 (6)	0.0043 (6)	0.0025 (6)
C6	0.0369 (9)	0.0229 (8)	0.0310 (8)	0.0010 (7)	0.0145 (7)	0.0022 (6)
C7	0.0366 (9)	0.0261 (8)	0.0244 (8)	0.0021 (7)	0.0082 (7)	0.0027 (6)
C8	0.0317 (8)	0.0211 (7)	0.0233 (7)	0.0022 (6)	0.0061 (6)	−0.0002 (6)
C9	0.0331 (9)	0.0291 (8)	0.0211 (7)	0.0020 (7)	0.0006 (6)	0.0015 (6)
C10	0.0266 (8)	0.0290 (8)	0.0226 (7)	0.0003 (6)	−0.0006 (6)	−0.0008 (6)
C5	0.0272 (8)	0.0284 (8)	0.0343 (9)	−0.0007 (7)	0.0090 (7)	0.0020 (7)
C11	0.0265 (8)	0.0213 (7)	0.0215 (7)	−0.0006 (6)	0.0040 (6)	−0.0010 (6)
C12	0.0233 (7)	0.0206 (7)	0.0222 (7)	0.0006 (6)	0.0002 (6)	−0.0008 (6)
C13	0.0247 (8)	0.0185 (7)	0.0247 (7)	−0.0009 (6)	0.0038 (6)	0.0002 (6)
C14	0.0228 (8)	0.0340 (9)	0.0319 (8)	0.0010 (7)	0.0035 (7)	0.0073 (7)
C15	0.0311 (9)	0.0308 (9)	0.0289 (8)	0.0011 (7)	0.0012 (7)	0.0086 (7)
C16	0.0317 (9)	0.0289 (8)	0.0272 (8)	−0.0033 (7)	0.0069 (7)	0.0057 (6)
C17	0.0240 (8)	0.0412 (10)	0.0325 (9)	−0.0022 (7)	0.0051 (7)	0.0056 (7)
C18	0.0235 (8)	0.0341 (9)	0.0260 (8)	−0.0004 (6)	0.0007 (6)	0.0048 (6)
C19	0.0225 (7)	0.0245 (7)	0.0185 (7)	−0.0001 (6)	0.0026 (6)	0.0036 (5)
C20	0.0230 (8)	0.0324 (9)	0.0267 (8)	−0.0009 (6)	0.0070 (6)	0.0007 (6)
C21	0.0222 (8)	0.0349 (9)	0.0307 (8)	−0.0053 (7)	0.0043 (6)	−0.0019 (7)
C22	0.0285 (8)	0.0261 (8)	0.0223 (7)	−0.0025 (6)	0.0043 (6)	−0.0003 (6)
C23	0.0237 (8)	0.0301 (8)	0.0232 (7)	0.0014 (6)	0.0058 (6)	0.0021 (6)
C24	0.0210 (7)	0.0280 (8)	0.0230 (7)	−0.0014 (6)	0.0022 (6)	0.0029 (6)

### Geometric parameters (Å, °)

**Table:**

Br—C6	1.902 (2)	C5—H5	0.9500
S—C1	1.756 (2)	C12—C13	1.461 (2)
S—C19	1.778 (2)	C13—C18	1.399 (2)
Cl—C22	1.741 (2)	C13—C14	1.402 (2)
O—C11	1.367 (2)	C14—C15	1.383 (2)
O—C12	1.379 (2)	C14—H14	0.9500
C1—C12	1.370 (2)	C15—C16	1.384 (2)
C1—C2	1.444 (2)	C15—H15	0.9500
C2—C11	1.383 (2)	C16—C17	1.385 (2)
C2—C3	1.421 (2)	C16—H16	0.9500
C3—C4	1.414 (2)	C17—C18	1.385 (2)
C3—C8	1.431 (2)	C17—H17	0.9500
C4—C5	1.363 (2)	C18—H18	0.9500
C4—H4	0.9500	C19—C20	1.390 (2)
C6—C7	1.366 (2)	C19—C24	1.391 (2)
C6—C5	1.401 (2)	C20—C21	1.384 (2)
C7—C8	1.414 (2)	C20—H20	0.9500
C7—H7	0.9500	C21—C22	1.387 (2)
C8—C9	1.430 (2)	C21—H21	0.9500
C9—C10	1.363 (2)	C22—C23	1.384 (2)
C9—H9	0.9500	C23—C24	1.387 (2)
C10—C11	1.402 (2)	C23—H23	0.9500
C10—H10	0.9500	C24—H24	0.9500
			
C1—S—C19	102.09 (7)	O—C12—C13	114.55 (13)
C11—O—C12	106.90 (12)	C18—C13—C14	118.09 (15)
C12—C1—C2	107.21 (13)	C18—C13—C12	119.32 (14)
C12—C1—S	126.72 (12)	C14—C13—C12	122.53 (14)
C2—C1—S	126.02 (12)	C15—C14—C13	120.62 (15)
C11—C2—C3	119.11 (14)	C15—C14—H14	119.7
C11—C2—C1	104.92 (14)	C13—C14—H14	119.7
C3—C2—C1	135.96 (14)	C14—C15—C16	120.68 (15)
C4—C3—C2	124.52 (14)	C14—C15—H15	119.7
C4—C3—C8	118.44 (15)	C16—C15—H15	119.7
C2—C3—C8	117.04 (14)	C15—C16—C17	119.32 (16)
C5—C4—C3	121.19 (15)	C15—C16—H16	120.3
C5—C4—H4	119.4	C17—C16—H16	120.3
C3—C4—H4	119.4	C18—C17—C16	120.50 (16)
C7—C6—C5	121.77 (16)	C18—C17—H17	119.7
C7—C6—Br	120.34 (13)	C16—C17—H17	119.7
C5—C6—Br	117.89 (13)	C17—C18—C13	120.78 (15)
C6—C7—C8	119.65 (15)	C17—C18—H18	119.6
C6—C7—H7	120.2	C13—C18—H18	119.6
C8—C7—H7	120.2	C20—C19—C24	120.00 (15)
C7—C8—C9	120.21 (15)	C20—C19—S	118.20 (12)
C7—C8—C3	119.26 (15)	C24—C19—S	121.76 (12)
C9—C8—C3	120.53 (15)	C21—C20—C19	120.17 (15)
C10—C9—C8	121.84 (15)	C21—C20—H20	119.9
C10—C9—H9	119.1	C19—C20—H20	119.9
C8—C9—H9	119.1	C20—C21—C22	119.34 (15)
C9—C10—C11	116.57 (15)	C20—C21—H21	120.3
C9—C10—H10	121.7	C22—C21—H21	120.3
C11—C10—H10	121.7	C23—C22—C21	121.09 (15)
C4—C5—C6	119.68 (16)	C23—C22—Cl	119.07 (12)
C4—C5—H5	120.2	C21—C22—Cl	119.84 (12)
C6—C5—H5	120.2	C22—C23—C24	119.38 (15)
O—C11—C2	111.08 (13)	C22—C23—H23	120.3
O—C11—C10	124.03 (14)	C24—C23—H23	120.3
C2—C11—C10	124.88 (15)	C23—C24—C19	119.98 (14)
C1—C12—O	109.87 (13)	C23—C24—H24	120.0
C1—C12—C13	135.35 (14)	C19—C24—H24	120.0
			
C19—S—C1—C12	96.64 (14)	C9—C10—C11—O	−178.71 (14)
C19—S—C1—C2	−86.08 (14)	C9—C10—C11—C2	1.5 (2)
C12—C1—C2—C11	1.00 (17)	C2—C1—C12—O	−1.22 (17)
S—C1—C2—C11	−176.71 (11)	S—C1—C12—O	176.48 (10)
C12—C1—C2—C3	−177.97 (16)	C2—C1—C12—C13	172.70 (16)
S—C1—C2—C3	4.3 (3)	S—C1—C12—C13	−9.6 (3)
C11—C2—C3—C4	−179.42 (14)	C11—O—C12—C1	0.94 (16)
C1—C2—C3—C4	−0.6 (3)	C11—O—C12—C13	−174.37 (12)
C11—C2—C3—C8	0.9 (2)	C1—C12—C13—C18	−162.13 (17)
C1—C2—C3—C8	179.78 (16)	O—C12—C13—C18	11.6 (2)
C2—C3—C4—C5	−179.47 (15)	C1—C12—C13—C14	15.1 (3)
C8—C3—C4—C5	0.2 (2)	O—C12—C13—C14	−171.21 (14)
C5—C6—C7—C8	1.5 (2)	C18—C13—C14—C15	1.2 (2)
Br—C6—C7—C8	−177.91 (11)	C12—C13—C14—C15	−176.09 (15)
C6—C7—C8—C9	179.43 (15)	C13—C14—C15—C16	−0.8 (3)
C6—C7—C8—C3	−0.5 (2)	C14—C15—C16—C17	−0.1 (3)
C4—C3—C8—C7	−0.4 (2)	C15—C16—C17—C18	0.6 (3)
C2—C3—C8—C7	179.30 (13)	C16—C17—C18—C13	−0.2 (3)
C4—C3—C8—C9	179.72 (14)	C14—C13—C18—C17	−0.7 (2)
C2—C3—C8—C9	−0.6 (2)	C12—C13—C18—C17	176.65 (15)
C7—C8—C9—C10	−179.19 (15)	C1—S—C19—C20	145.84 (13)
C3—C8—C9—C10	0.7 (2)	C1—S—C19—C24	−36.64 (14)
C8—C9—C10—C11	−1.1 (2)	C24—C19—C20—C21	−1.4 (2)
C3—C4—C5—C6	0.8 (2)	S—C19—C20—C21	176.17 (13)
C7—C6—C5—C4	−1.7 (3)	C19—C20—C21—C22	0.5 (3)
Br—C6—C5—C4	177.73 (12)	C20—C21—C22—C23	1.2 (3)
C12—O—C11—C2	−0.27 (16)	C20—C21—C22—Cl	−178.21 (13)
C12—O—C11—C10	179.87 (14)	C21—C22—C23—C24	−2.0 (2)
C3—C2—C11—O	178.73 (12)	Cl—C22—C23—C24	177.39 (12)
C1—C2—C11—O	−0.45 (17)	C22—C23—C24—C19	1.1 (2)
C3—C2—C11—C10	−1.4 (2)	C20—C19—C24—C23	0.6 (2)
C1—C2—C11—C10	179.41 (14)	S—C19—C24—C23	−176.92 (12)
